# The impact of infectious disease prevention behavior on quality of life: A moderated mediation model

**DOI:** 10.1002/hcs2.11

**Published:** 2022-09-07

**Authors:** Fei Wang, Pu Ge, Danyang Li, Lin Cai, Xialei Li, Xinying Sun, Yibo Wu

**Affiliations:** ^1^ State Key Laboratory of Cognitive Neuroscience and Learning Beijing Normal University Beijing China; ^2^ Institute of Chinese Medical Sciences & State Key Laboratory of Quality Research in Chinese Medicine University of Macau Macau China; ^3^ Medical College Xi'an Peihua University Xi'an China; ^4^ School of Marxism Sichuan Institute of Industrial Technology Deyang China; ^5^ School of Pharmaceutical Sciences Shandong University Jinan China; ^6^ School of Public Health Peking University Beijing China

**Keywords:** family structure, infectious disease prevention behavior, quality of life, self‐efficacy

## Abstract

**Objective:**

To explore the mechanism of infectious disease prevention behavior on quality of life, and to investigate the mediating role of self‐efficacy and the moderating role of family structure.

**Methods:**

A total of 3015 subjects were selected by multistage stratified cluster sampling.

**Results:**

Infectious disease prevention behavior had a significant positive predictive effect on the quality of life (*β* = 0.08, *p* < 0.001), The self‐efficacy of family members had a partial mediating effect on the relationship between infectious disease prevention behavior and quality of life (*β* = 0.01, *p* < 0.001). Compared to nuclear family, conjugal family (*β* = 0.05, *p* < 0.001) and single‐parent family (*β* = 0.04, *p* < 0.01) could regulate the relationship between infectious disease prevention behavior and the quality of life, stem family (*β* = −1.53, *p* < 0.05), conjugal family (*β* = 1.63, *p* < 0.05), and collective family (*β* = −1.37, *p* < 0.05) could regulate the relationship between infectious disease prevention behavior and self‐efficacy, conjugal family (*β* = 0.00, *p* < 0.001) could regulate the relationship between self‐efficacy and quality of life.

**Conclusion:**

Infectious disease prevention behavior can affect the quality of life through self‐efficacy. Different family structures play a regulatory role in different paths, and a regulatory mediation model is established.

## Graphical abstract

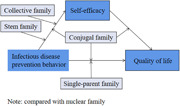

**Abbreviations**
AC: alcohol consumption
ColF: collective family
ConF: conjugal family
EL: education level
FS: family Structure
HI: household income
IDPB: infectious disease prevention behavior
MS: marital status
NF: nuclear family
PCM: per capita monthly
QOL: quality of life
SE: self‐efficacy
SF: stem family
SPF: single‐parent family
SS: smoking status

## 1 INTRODUCTION

Since December 2019, the COVID‐19 has been ravaging the world, posing a serious threat to the lives of people worldwide. With the implementation of effective prevention and treatment measures by the Chinese government, the epidemic situation in China has improved and this respiratory infectious disease has been brought under control. Therefore, more and more people realize the importance of infectious disease prevention behavior. Although a large body of research has confirmed that infectious disease prevention behavior can significantly improve the quality of life of individuals [1], the mechanisms of infectious disease prevention behavior affecting the quality of life are not clear. We provide possible mechanisms by which infectious disease prevention behaviors influence the quality of life, such as self‐efficacy and family structure.

### 1.1 Infectious disease prevention behavior, self‐efficacy, and the quality of life

Health Self‐Empowerment Theory (HSET) emphasizes the importance of individual cognitive‐behavioral variables (e.g., health motivation, health self‐efficacy, positive coping) in promoting people's health [2]. As a specific type of positive coping, infectious disease prevention behavior has been found to play a significant role in health promotion [3]. Infectious disease prevention behavior refers to a range of health prevention behaviors taken by individuals to prevent and treat infectious diseases, such as choosing to wear a mask outside during the outbreak to prevent respiratory diseases. Some researchers have found that infectious disease prevention behavior is very important for improving the quality of life of patients with primary immunodeficiency diseases [4] and is an important measure in ensuring healthy aging and maintaining the quality of life [5]. Infectious disease prevention behavior not only affects the individual's quality of life but also affects the individual's sense of self‐efficacy [6].

Self‐efficacy was first proposed by Bandura. It refers to people's confidence in whether they can use their own skills to complete a certain work behavior, and its formation is influenced by the experience of success or failure in the behaviors [7]. Infectious disease prevention behavior as a successful behavior to prevent infection is bound to increase the individual's sense of self‐efficacy. In studies of specific infectious diseases, a strong correlation has been found between infectious disease prevention behavior (e.g., vaccination) and an individual's sense of self‐efficacy [8], such that those who choose to wear a mask during the outbreak clearly know that the outcome of their behavior for themselves is a reduced probability of illness, further enhancing self‐efficacy. As a positive developmental indicator, self‐efficacy has a positive effect on the quality of life [3] and has been found to be a strong predictor of the individual's quality of life [9]. In studies on patients with epilepsy [10] and severe myasthenia gravis [11], the predictive effect of self‐efficacy on patients' quality of life has been confirmed.

### 1.2 The regulatory role of family structure

According to Ecological Systems Theory, a developing individual is subject to four environmental systems—micro, intermediate, outer, and macro—which influence many aspects of individual development [12]. As the core part of the microsystem, the family has a direct effect on individual development and outcomes. The family structure in which people usually live is a structural determinant of health [13], and each family can have different family structures, such as the nuclear family (family consisting of parents and unmarried children), stem family (family consisting of parents and a married pair of children), conjugal family (family consisting of parents only), collective family (family consisting of several unrelated people living together temporarily), and single‐parent family (family consisting of divorced or widowed persons with children), and so forth. In a family study of children with chronic epilepsy, family structure type was found to be prominently associated with self‐efficacy [14]. Compared with the self‐efficacy of members with an incomplete family structure, the self‐efficacy of members with an intact family structure was better [15]. Previous studies have found that infectious disease prevention behavior can significantly enhance individuals' self‐efficacy [16], and different family structures can also affect individuals' self‐efficacy, which means that there may be differences in the impact of infectious disease prevention behavior on individuals' self‐efficacy in families that may vary across family structures.

In addition, it has been established that family structure is a structural driver of family members' health [17]. Martins et al.'s [18] study showed that older people living with their children had poorer quality of life outcomes, while Liu et al. [19] found that compared to those with children, the quality of life of the elderly without children is significantly reduced. We found that older people living with their children had access to family social support and were predictive of older people's health status [20]. This illustrates the inconsistency in findings obtained so far in research on the relationship between core families, conjugal family structure, and the quality of life of family members. Some studies have suggested that family relationship is also a factor affecting the quality of life of older people [21]; therefore, the marital status of children is bound to affect the quality of life of parents. To avoid the factor of children's marital status from interfering with the experimental results, this study further differentiated the family structure of parents living with their children into nuclear and stem families, and defined the family structure of parents living alone as a conjugal family. Compared to previous studies, the family structure of Chinese society has undergone significant changes due to the implementation of family planning [22]. With more collective family structures, it is still unknown whether there are differences in the quality of life of collective family members compared to traditional nuclear family. Unlike the intact family mentioned above, the single‐parent family has received increasing attention from researchers as a specific family structure, and it has been well documented that single‐parent family members will face increased behavioral and emotional problems [23].

### 1.3 The research model

**Figure 1:**
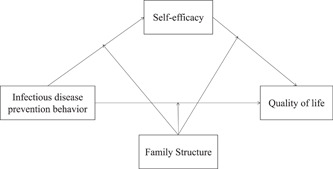
The proposed theoretical model.

To put them together, the relationship model proposed earlier is shown in Figure 1. Through a moderated mediation model, we (a) tested whether self‐efficacy mediated the relationship between infectious disease prevention behavior and the quality of life to some extent, and (b) whether different kinds of family structures played a regulatory role between infectious disease prevention behavior and self‐efficacy, between self‐efficacy and the quality of life, and between infectious disease prevention behavior and the quality of life.

In general, the mechanism of the impact of infectious disease prevention behavior on quality of life is still unclear, and whether family structure plays a role in this process is still unclear. Therefore, the model constructed in this study is conducive to reveal the mechanism of infectious disease prevention behavior affecting the quality of life from the perspective of family structure, and lay a theoretical foundation for the improvement of quality of life in the future.

## 2 OBJECTS AND METHODS

### 2.1 Participants

A total of 3218 permanent residents in 28 cities across China were selected from October 2020 to December 2020, and the respondents were involved in various age groups and social strata.

### 2.2 Methods

#### 2.2.1 Sampling method

Using a multistage stratified cluster sampling method, based on geographical division and the distribution of the population, a total of 14 provinces (autonomous regions and municipalities directly under the Central Government) were selected from each of the seven administrative regions of Eastern China, Southern China, Northern China, Central China, Southwest China, Northeast China, and Northwest China with the random number table method, that is, Shandong Province and Jiangsu Province in Eastern China; Guangdong Province and Hainan Province in Southern China; Beijing City, Inner Mongolia Autonomous Region, and Shanxi Province in Northern China; Henan Province and Hunan Province in Central China; Sichuan Province and Chongqing City in Southwest China; Liaoning Province and Heilongjiang Province in Northeast China; Shanxi Province and Xinjiang Uygur Autonomous Region in Northwest China. Then two cities were selected from each of the selected provinces using the random number table method, skipping this step if they were municipalities directly under the Central Government. Following this, the sample distribution of each age group was made to basically match the demographic characteristics based on the age distribution of China's population. Finally, the urban−rural population distribution of the entire sample was restricted so that it generally matched the urban−rural population ratio in China. One investigator was recruited in each city and, after systematic training, each investigator was responsible for returning a range of 80−120 questionnaires.

#### 2.2.2 Instruments

##### New General Self‐Efficacy Scale

The NGSES developed by Chen et al. [24] and revised by Xiao and Yi [25] was adopted. The scale consists of eight questions and is scored using Likert 5‐level scoring (1 for *strongly disagree* and 5 for *strongly agree*), with higher scale scores indicating higher individuals' self‐efficacy. The internal consistency coefficient of the scale in this study was 0.96.

##### Quality of Life Questionnaire

The Europe Quality five‐dimensional five‐level questionnaire (EQ‐5D‐5L) is a universal health status measurement instrument and its reliability and validity have been confirmed by many studies [26]. The scale is composed of five dimensions of mobility, self‐care, anxiety or depression, daily activities (e.g., work, study, housework, family, or leisure activities), pain or discomfort, and visual analog scale (EQ‐VAS). The utility index values of the first component will be used to measure the quality of life of the subjects in this study.

##### Self‐Compiled Infectious Disease Prevention Behavior Questionnaire

According to the relevant descriptions of the Healthy China Initiative (2019−2030) planning outline regarding actions to prevent and treat infectious and endemic diseases, a questionnaire on infectious disease prevention behavior was developed with three items, namely covering the mouth and nose with arms or tissues when coughing and sneezing, actively vaccinating, and avoiding contact with sick animals and poultry. The options are based on a cross‐theoretical model design, including “do not intend to take the behavior” (unintentional period), “intend to take the behavior but have not decided when to start” (intentional period), “decide to take the behavior soon or immediately” (preparatory period), ”start to try to take the behavior” (trial period), “have taken the behavior but have not persisted in it for a long time (less than six months)” (action period), and “have persisted for a long time (more than six months)” (maintenance period), which are scored as 0, 1, 2, 3, 4, and 5 respectively. In this study, the split‐half reliability of the questionnaire was 0.73 and the Cronbach's *α* coefficient was 0.79.

##### Self‐compiled family structure questionnaire

The questionnaire was designed to distinguish different family structures by measuring the recent family members of those who completed it, namely nuclear family (family consisting of parents and unmarried children), stem family (family consisting of parents and a married pair of children), conjugal family (family consisting of parents only), collective family (family consisting of several unrelated people living together temporarily), and single‐parent family (family formed by divorced or widowed persons with children). There are two items, of which Item 2 is a multiple‐choice question. The questions are 1. What is your marital status? Unmarried, married, divorced, widowed; 2. Who has lived with you in the last two months (if so, please fill in how many people)? Spouse, own father, own mother, spouse's father, spouse's mother, siblings, sons, daughters, nanny, dormitory housemates, and others.

#### 2.2.3 Quality control

The investigators recruited in each city were highly educated and systematically trained. Consistent instructions were used and subjects completed the questionnaire on their own. For those who could not complete it independently, the investigators read it to the subjects one by one, asked for their specific comments, and then assisted them in completing it. On‐site verification was carried out after completion, and gaps were filled in during communication with the subjects. Questionnaires with inconsistent logic and those that took less than 120 s to complete were screened out after recall.

#### 2.2.4 Statistical analysis

To test the correlation between the variables, we used Spss22.0 to conduct Pearson correlation analysis, where a correlation coefficient of <0.30 is generally considered to be low, 0.30−0.60 is moderate, and >0.60 is high when the correlation is significant [27]. As this study used a questionnaire survey, to exclude the possible problem of common method bias in the study, we used Amos 22.0 to construct a one‐way structural equation model and judged whether there was a more serious problem of common method bias by comparing the difference in fit indices between the one‐way model and the original model, and if the fit of the constructed one‐way model was much worse than the original model, that is, it can be concluded that the study does not have a more serious common method bias problem [28]. To further test the validity of the hypothesis model, we constructed the model using the process plug‐in developed by Hayes. First, to assess the moderating effect of family structure (the moderating variable) on infectious disease prevention (the independent variable) and quality of life (the dependent variable), we used Model 1 from Process to test whether the moderating effect between the moderating variable and the independent variable was significant [29]. Second, we used Model 1 to test the moderating effect of family structure (the moderating variable) between infectious disease prevention (the independent variable) and self‐efficacy (the mediator variable). Finally, following the causal step approach, to test the validity of the mediator model with moderation, we used Model 3 to test the prediction of self‐efficacy (the mediator variable) on quality of life (the dependent variable) and the prediction of family structure (the moderating variable) on the posterior of the model. The moderating effect of the moderating variable in the second half of the model is significant if the interaction term is significant, while the mediating effect is significant if the effect of the independent variable on the mediator variable is significant and the effect of the mediator variable on the dependent variable is significant according to the causal step approach [29].

## 3 RESULTS

### 3.1 Common method biases test

According to a related study [28], for possible common method bias in this study, the confirmatory factor analysis was conducted to test for common method bias for all scale questions, and the results showed that the model fitting was very poor. *χ* ^2^/*df* = 118.79, comparative fit index = 0.72, goodness‐of‐fit index = 0.59, adjusted goodness‐of‐fit index = 0.47, normed fit index = 0.71, and Root Mean Square Error of Approximation = 0.20, so there is no serious common method bias problem.

### 3.2 Correlation analysis of each variable

The results of Pearson's correlation analysis are presented in Table 1. Setting the categorical variable of family structure as a dummy variable and analyzing the nuclear family group as a control, some moderate to high significant correlations were found between infectious disease prevention behavior, self‐efficacy, and the quality of life; compared to the nuclear family, only conjugal families were significantly correlated with infectious disease prevention behavior, self‐efficacy, and the quality of life; compared to the nuclear family, the single‐parent family was significantly correlated with the quality of life.

**Table 1:** Results of correlation analysis

	1	2	3	4	5	6	7
1. Infectious disease prevention behavior	1						
2. Self‐efficacy	0.44***	1					
3. Quality of life	0.43***	0.61***	1				
4. Nuclear family	0.03	0.07***	0.02	1			
5. Conjugal family	−0.06**	−0.04*	−0.10***	−0.18***	1		
6. Collective family	−0.01	−0.10**	0.00	−0.28***	−0.31***	1	
7. Single‐parent family	0.01	−0.03	−0.05*	−0.11***	−0.12***	−0.19***	1

* Significantly correlated at 0.05 level.

** Significantly correlated at 0.01 level.

*** Significantly correlated at 0.001 level.

### 3.3 Tests of moderated mediators

Wen and Ye [30] believed that three equations should be constructed to test the effect of the moderated mediation model, where Equation (1) estimated the moderating effect of the moderating variable (family structure) on the independent variable (infectious disease prevention) and the dependent variable (quality of life); Equation (2) estimated the moderating effect of the moderating variable (family structure) on the independent variable (infectious disease prevention) and the mediator variable (self‐efficacy), and Equation (3) estimated the moderating effect of the moderating variable (family structure) on the mediator variable (self‐efficacy) and the dependent variable (the quality of life) and the influence of the residual independent variable on the dependent variable. The results are presented in Table 2. In Equation (1), the main effect of infectious disease prevention was significant (*β* = 0.08, *t* = 12.20, *p* < 0.001), with conjugal family (*β* = −0.06, *t* = −4.10, *p* < 0.001), collective family (*β* = −0.04, *t* = −3.18, *p* < 0.01), and single‐parent family (*β* = −0.08, *t* = −4.18, *p* < 0.001) had significantly lower quality of life than the nuclear family, compared to the nuclear family, and only conjugal family (*β* = 0.05, *t* = 4.65, *p* < 0.001) and single‐parent family (*β* = 0.04, *t* = 2.73, *p* < 0.01) had significant interactions with infectious disease prevention, that is, the moderating effect of family structure on infectious disease prevention and the quality of life was significant in conjugal family and single‐parent family. In Equation (2), the main effect of infectious disease prevention was significant (*β* = 6.14, *t* = 14.99, *p* < 0.001), with conjugal family (*β* = −3.04, *t* = −3.17, *p* < 0.01), collective family (*β* = −3.33, *t* = −3.91, *p* < 0.001), and single‐parent family (*β* = −4.66, *t* = −3.71, *p* < 0.001) had significantly lower self‐efficacy than nuclear family, compared to the nuclear family, and the interaction between stem family (*β* = −1.53, *t* = −2.11, *p* < 0.05), conjugal family (*β* = 1.63, *t* = 2.52, *p* < 0.05) and collective family (*β* = −1.37, *t* = −2.45, *p* < 0.05) and infectious disease prevention was significant, that is, the moderating effect of family structure on infectious disease prevention and self‐efficacy was significant in stem family, conjugal family, and collective family. In Equation (3), the main effects of infectious disease prevention (*β* = 0.04, *t* = 11.51, *p* < 0.001) and self‐efficacy (*β* = 0.01, *t* = 17.54, *p* < 0.001) were significant, with conjugal family (*β* = −0.04, *t* = −2.96, *p* < 0.01) and single‐parent family (*β* = −0.04, *t* = −2.59, *p* < 0.01) had significantly lower quality of life than nuclear family, compared to nuclear family, and only the interaction between stem family (*β* = 0.00, *t* = 5.82, *p* < 0.001) and infectious disease prevention was significant, that is, in stem family, the moderating effect of family structure on infectious disease prevention and self‐efficacy was significant.

**Table 2:** Results of the model tests

	Equation (1) Dependent variable: quality of life		Equation (2) Dependent variable: self‐efficacy		Equation (3) Dependent variable: quality of life	
	*β*	*t*	*β*	*t*	*β*	*t*
Infectious disease prevention *X*	0.08	12.20***	6.14	14.99***	0.04	11.51***
Stem family *W*1	−0.02	−0.99	−0.04	−0.04	−0.02	−1.29
Conjugal family *W*2	−0.06	−4.10***	−3.04	−3.17**	−0.04	−2.96**
Collective family *W*3	−0.04	−3.18**	−3.33	−3.91***	−0.02	−1.56
Single‐parent family *W*4	−0.08	−4.18***	−4.66	−3.71***	−0.04	−2.59**
*X *× *W*1	−0.02	−1.40	−1.53	−2.11*		
*X *× *W*2	0.05	4.65***	1.63	2.52*		
*X *× *W*3	−0.00	−0.03	−1.37	−2.45*		
*X *× *W*4	0.04	2.73**	0.93	0.98		
Self‐efficacy *M*					0.01	17.54***
*M *× *W*1					0.00	0.77
*M *× *W*2					0.00	5.82***
*M *× *W*3					−0.00	−0.25
*M *× *W*4					0.00	1.68
Gender	0.02	1.52	−1.79	−2.68**	0.03	3.27**
Education level	0.00	0.68	−0.13	−0.52	0.00	1.06
Marital status	−0.01	−0.40	1.98	2.39*	−0.02	−1.79
Alcohol consumption	−0.00	−0.79	−0.89	−3.04**	0.00	0.85
Smoking status	−0.01	−1.08	−0.62	−1.03	−0.01	−0.69
Age	−0.00	−0.72	0.08	0.19	−0.01	−1.05
Per capita monthly household income	−0.01	−1.53	0.06	0.24	−0.01	−1.83
*R* ^2^	0.21		0.22		0.43	
*F*	49.92***		53.62***		132.87***	

* Significantly correlated at the 0.05 level.

** Significantly correlated at the 0.01 level.

*** Significantly correlated at the 0.001 level.

According to the above three equations, self‐efficiency plays an intermediary role between infectious diseases prevention and quality of life; Family structure plays a moderating role in the path, specifically compared with the nuclear family, only the conjugal family mediates the first and second half of the “infectious diseases prevention → self‐efficacy → quality of life” and also the direct effect of infectious diseases prevention, the single‐parent family only mediates the direct effect of infectious diseases prevention on the quality of life, and the collective family and the stem family mediate the first half of the “infectious diseases prevention → self‐efficacy → quality of life.”

**Figure 2:**
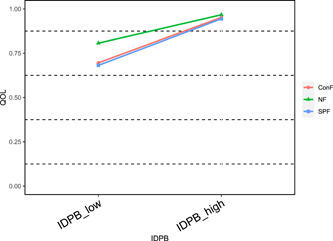
Moderation of infectious disease prevention behavior and quality of life by family structure (insignificant removed). ConF, conjugal family; IDPB, infectious disease prevention behavior; NF, nuclear family; QOL, quality of life; SPF, single‐parent family.

**Figure 3:**
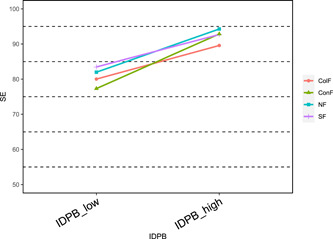
Moderation of family structure on infectious disease prevention and self‐efficacy (insignificant removed). ColF, collective family; ConF, conjugal family; IDPB, infectious disease prevention behavior; NF, nuclear family; SE, self‐efficacy; SF, stem family.

**Figure 4:**
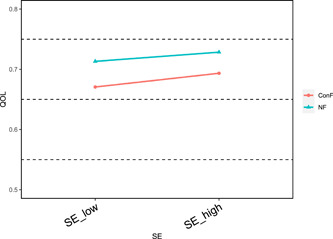
Moderation of family structure on self‐efficacy and quality of life (insignificant removed). ConF, conjugal family; NF, nuclear family; QOL, quality of life; SE, self‐efficacy.

To better understand the moderating effect of family structure, a further simple slope analysis was conducted to distinguish between high and low independent variables by means plus or minus one standard deviation. The results showed that on the pathway “Infectious disease prevention → quality of life,” compared to nuclear family (*β* = 0.08, *t* = 12.20, *p* < 0.001), both conjugal family (*β* = 0.13, *t* = 16.00, *p* < 0.001) and single‐parent family (*β* = 0.12, *t* = 8.91, *p* < 0.001) significantly enhanced the effect of infectious disease prevention on the quality of life (see Figure 2); in the first half of the pathway “Infectious disease prevention → self‐efficacy → quality of life,” compared to nuclear family (*β* = 6.14, *t* = 14.99, *p* < 0.001), stem family (*β* = 4.61, *t* = 7.65, *p* < 0.001) and collective family (*β* = 4.77, *t* = 12.46, *p* < 0.001) significantly attenuated the enhancing effect of infectious disease prevention on self‐efficacy, while conjugal family (*β* = 7.77, *t* = 15.54, *p* < 0.001) significantly enhanced the enhancing effect of infectious disease prevention on self‐efficacy (see Figure 3); in the second half of the pathway “Infectious disease prevention → self‐efficacy → quality of life,” compared to nuclear family (*β* = 0.008, *t* = 17.54, *p* < 0.001), conjugal family (*β* = 0.011, *t* = 21.79, *p* < 0.001) significantly enhanced the enhancing effect of self‐efficacy on quality of life (see Figure 4).

## 4 DISCUSSION

### 4.1 The relationship between infectious disease prevention behavior and the quality of life

Studies have found significant predictive effects of infectious disease prevention behaviors on quality of life. It has been found that the global pandemic of COVID‐19 has severely affected people's quality of life [31], and an individual's infectious disease prevention behavior can enhance his quality of life when an individual's infectious disease prevention behavior is higher, the quality of life is also higher [32]. The present study supports the findings of previous studies, the reason may be: have an infectious disease itself can severely impair an individual's quality of life, whereas for those who are not infected with an infectious disease, the fear of becoming ill can seriously threaten their quality of life, and infectious disease prevention behaviors can enhance their perceived quality of life by reducing their perceived risk of becoming ill to varying degrees.

### 4.2 The mediating role of self‐efficacy

The results of the study revealed a significant mediating role of self‐efficacy in that infectious disease prevention behavior further influenced the quality of life by affecting individuals' self‐efficacy, and the more infectious disease prevention behaviors individuals engaged in, the higher their self‐efficacy and the higher their quality of life scores. This confirms the research hypothesis (a): Self‐efficacy theory points out that the formation of self‐efficacy is mainly influenced by five factors, including the experience of success or failure of behavior, alternative experience, verbal persuasion, emotional arousal, and situational conditions [7]. Experience of success or failure of behavior refers to the information or direct experience gained through manipulation. Successful experience can increase an individual's sense of self‐efficacy and give the individual confidence in their own behavior. This successful experience in infectious disease prevention behavior, which reduces the risk of contracting infectious diseases and ensures the quality of life, increases an individual's self‐efficacy and further enhances the quality of life. In addition to this, the alternative experience of success from others, as a result of the mitigation of the COVID‐19 outbreak due to the implementation of infectious disease prevention measures, also enhances individuals' self‐efficacy and further influences quality of life. A strong correlation has been found between infectious disease prevention behaviors (e.g., vaccination) and individuals' self‐efficacy [33], such that those who choose to wear a mask during the outbreak are clearly aware that their behaviors will lead to a decrease in the probability of becoming ill, that is, an increase in self‐efficacy. Self‐efficacy, in turn, is a strong predictor of quality of life [34], that is, infectious disease prevention behavior can lead to family members feeling less at risk of contracting infectious diseases, which can lead to an increase in their self‐efficacy and, ultimately, their quality of life.

### 4.3 The impact of infectious disease prevention behavior on quality of life: The moderating role of family structure

Ecosystem theory suggests that the developing individual is influenced by the family within the microsystem [12], and within the family which people depend on for survival, family structure has been found to be a structural determinant of the health of family members [35]. In contrast to the most common nuclear family structure, different family structures can have different effects on the quality of life of family members.

The results of this study indicate that the direct predictive effect of infectious disease prevention behavior on the quality of life of family members was more pronounced in conjugal and single‐parent families than in the nuclear family. It should be noted that this moderating pattern does not imply that nonintegral family such as a single‐parent family has a more positive effect on the quality of life than an intact family such as a nuclear family. The reason for this may be that the quality of life in a nuclear family is already at a high level at the time of low infectious disease prevention behavior when the improvement in quality of life is more constrained by factors other than infectious disease prevention behaviors, and therefore the effect of infectious disease prevention behavior on quality of life is significantly less pronounced in the nuclear family than in the conjugal and single‐parent family. Based on this result, it is important to focus on strengthening infectious disease prevention behavior in a conjugal and single‐parent family, as infectious disease prevention behavior is highly effective in improving the quality of life in these two collectives of families.

Previous research has found that members of nonintegral family structures have lower self‐efficacy compared to members of intact family structures [15], and similar findings were reached in this study: that is, compared to a nuclear family, single‐parent family significantly negatively predicted self‐efficacy. Furthermore, it was found that compared to the nuclear family, the stem family and collective family significantly weakened the promotion effect of infectious disease prevention behavior on self‐efficacy, further affecting the quality of life; and compared to the nuclear family, infectious disease prevention behavior was more likely to lead to the improvement of self‐efficacy in conjugal family, and then improve the quality of life. This may be due to the fact that a collective family is a special form of family in which there are no blood ties and members from different regions have different cultural habits, which leads to the problem of adaptability of members in the collective family [36]. The presence of adaptability then weakens the promotion effect of infectious disease prevention behavior on self‐efficacy, further affecting the quality of life. In contrast, members of stem family structure each have their own lifestyles, and, compared to other families, are more likely to produce conflicts and disputes; such conflicts may have an impact on the healthy development of family members, and a series of conflicts such as mother‐in‐law−daughter‐in‐law relationships will weaken the promotion effect of infectious disease prevention behavior on self‐efficacy, which will further affect the self‐efficacy of family members. The biggest difference between a conjugal family and a nuclear family lies in the presence or absence of children. It has been found that with the birth of children, parents have a lot of invisible pressure and need to shoulder more responsibilities [37]. Such pressure will weaken the promotion effect of infectious disease prevention promotion on self‐efficacy and further affect the quality of life.

This study found that no matter the level of self‐efficacy, the nuclear family had higher quality of life than the conjugal family, which to some extent supports Liu et al.'s view that the quality of life of stem family members is higher than that of conjugal family members. The presence of children in a family can significantly increase family supportiveness [38], and family members quality of life depends on family support [39]; such a nuclear family would attach more importance to the quality of life of children and family members, which is likely to be the reason why the quality of life of a nuclear family is higher than that of conjugal family. In addition, although nuclear family members significantly outperformed conjugal family members in terms of quality of life, the effect of self‐efficacy on quality of life was enhanced in the conjugal family compared to the nuclear family. This result suggests that family structure moderates the relationship between self‐efficacy and quality of life. Previous research has found a strong relationship between family factors and self‐efficacy [40], with the self‐efficacy of nuclear family members being influenced by a range of variables such as parent−child relationships [13] and parenting styles [41], whereas the self‐efficacy of conjugal family members is not influenced by these variables. Thus, compared to the nuclear family, conjugal families are more sensitive to the effects of infectious disease prevention behavior, that is, family structure plays a moderating role in the relationship between self‐efficacy and the quality of life.

### 4.4 Research significance and deficiency

This study explored the impact and mechanisms of action of infectious disease prevention behavior on the quality of life of family members through a moderated mediation model. This study mainly found the mechanism of infectious disease prevention behavior affecting the quality of life, and also found the important role of different family structures. This study reveals the mechanism of infectious disease prevention behavior promoting quality of life from the perspective of family structure for the first time and provides theoretical support for improving the quality of life and self‐efficacy of different types of families in the future.

There are still defects and deficiencies in this study that need to be improved in future research. First, this study compared other family structures with the nuclear family and did not specifically analyze the differences between other family structures. Second, the infectious disease prevention behaviors in this study are more oriented toward the prevention of respiratory infectious diseases, and future research could further expand the concept of infectious disease prevention behavior to explore the impact of the prevention of infectious diseases such as AIDS on the quality of life.

## AUTHOR CONTRIBUTIONS

Fei Wang analyzed the data and prepared the first draft of the manuscript. Pu Ge and Danyang Li participated in the conception and design of the study, Lin Cai constructively revised the manuscript; Xialei Li participated in data collection and organization; Xinyin Sun and Yibo Wu participated in and supervised the study throughout, and they share corresponding authorship. All authors commented on previous versions of the manuscript and approved the final version.

## CONFLICT OF INTEREST

The authors declare no conflict of interest.

## ETHICS STATEMENT

This quantitative study was performed in accordance with the Ministry of Health “involves people of biomedical research ethics review method (try out)”, the national drug supervision and administration of the quality control standard for clinical trials (2003), the Medical Instrument Clinical Trial Regulations (2004), the Declaration of Helsinki. This study passed the ethical review (JKWH‐2020‐17). We certify that all applicable institutional and governmental regulations concerning the ethical use of human volunteers were followed over the course of this study. All interviewees provided written informed consent to participate in this study upon recruitment.

## INFORMED CONSENT

None.

## Data Availability

The data sets generated and/or analyzed during the current study are not publicly available because the data still need to be used for other research but are available from the corresponding author on reasonable request.

## References

[1] Hong PC , Chen KJ , Chang YC , Cheng SM , Chiang HH . Effectiveness of theory‐based health information technology interventions on coronary artery disease self‐management behavior: a clinical randomized waitlist‐controlled trial. J Nurs Scholarsh. 2021;53(4):418–27. 10.1111/jnu.12661 33844425 PMC8359962

[2] Tucker CM , Butler AM , Loyuk IS , Desmond FF , Surrency SL . Predictors of a health‐promoting lifestyle and behaviors among low‐income African American mothers and White mothers of chronically ill children. J Natl Med Assoc. 2009;101(2):103–10. 10.1016/s0027-9684(15)30821-x 19378625

[3] Wippold GM , Frary SG . The role of modifiable, self‐empowerment‐oriented variables to promote health‐related quality of life among inadequately insured Americans. J Prim Prev. 2021;43:95–110. 10.1007/s10935-021-00652-1 PMC8924987 34773547

[4] Ishimura M , Takada H , Doi T , Imai K , Sasahara Y , Kanegane H , et al. Nationwide survey of patients with primary immunodeficiency diseases in Japan. J Clin Immunol. 2011;31(6):968–76. 10.1007/s10875-011-9594-7 21956496

[5] Weinberger B . Vaccination of older adults: influenza, pneumococcal disease, herpes zoster, COVID‐19 and beyond. Immun Ageing. 2021;18(1):38. 10.1186/s12979-021-00249-6 34627326 PMC8501352

[6] Kim I‐O , 박현정. A . Survey on the situation. Experience and educational need of infectious diseases management of childcare teacher. Korean J Child Educ Care. 2014;14(1):23–50.

[7] Bandura A . Perceived self‐efficacy in the exercise of control over AIDS infection. Eval Program Plan. 1990;13(1):9–17. 10.1016/0149-7189(90)90004-G

[8] Yoo W , Choi DH , Park K . The effects of SNS communication: how expressing and receiving information predict MERS‐preventive behavioral intentions in South Korea. Comput Human Behav. 2016;62:34–43. 10.1016/j.chb.2016.03.058 32288174 PMC7127459

[9] Lo HHM . Quality of life among adolescents in Hong Kong: general and gender‐specific effects of self‐efficacy and mindfulness: a special issue on quality of life in Chinese societies. Appl Res Qual Life. 2021;16(6):2311–34. 10.1007/s11482-021-09914-w

[10] Lee SA , Kim SJ , Korean Qo LESG . Self‐efficacy in seizure management differentially correlated with quality of life in persons with epilepsy depending on seizure recurrence and felt stigma. Seizure—Eur J Epilep. 2020;81:91–5. 10.1016/j.seizure.2020.07.029 32771824

[11] Fan X , Xing CY , Yang L , Wang J , Feng LS . Fatigue, self‐efficacy and psychiatric symptoms influence the quality of life in patients with myasthenia gravis in Tianjin, China. J Clin Neurosci. 2020;79:84–9. 10.1016/j.jocn.2020.06.023 33070925

[12] Bronfenbrenner U . The ecology of human development: experiments by nature and design. Harvard University Press; 1979.

[13] Banik A , Zarychta K , Knoll N , Luszczynska A . Cultivation and enabling effects of social support and self‐efficacy in parent−child dyads. Ann Behav Med. 2021;55(12):1198–1210. 10.1093/abm/kaab004 33772544 PMC8601043

[14] Cui C , Li SZ , Zheng XL , Cheng WJ , Ting W . Participation in healthcare behavior by adolescents with epilepsy and factors that influence it during the transition period: a cross‐sectional study in China. Epilep Behav. 2020;113:113107576. 10.1016/j.yebeh.2020.107576 33232895

[15] Guo XM . The association between family structure and subjective well‐being among emerging adults in China: examining the sequential mediation effects of maternal attachment, peer attachment, and self‐efficacy. J Adult Dev. 2019;26(1):22–30. 10.1007/s10804-018-9293-1

[16] Yoo HJ , Song E . Effects of personal hygiene habits on self‐efficacy for preventing infection, infection‐preventing hygiene behaviors, and product‐purchasing behaviors. Sustainability. 2021;13(17):9483. 10.3390/su13179483

[17] Barrett AE , Turner RJ . Family structure and mental health: the mediating effects of socioeconomic status, family process, and social stress. J Health Soc Behav. 2005;46(2):156–69. 10.1177/002214650504600203 16028455

[18] Martins NPD , Silqueira SMD , Souza LME , Souza CDM , Soares SM , Matos SS . Quality of life of older adults admitted to a medical clinic unit of a public hospital in Brazil. Rev Esc Enferm Usp. 2020;54:54e03573. 10.1590/s1980-220x2018032903573 32696942

[19] Liu HJ , Han XH , Xiao QY , Li SZ , Feldman MW . Family structure and quality of life of elders in rural China: the role of the new rural social pension. J Aging Soc Pol. 2015;27(2):123–38. 10.1080/08959420.2014.977662 PMC5473658 25356822

[20] Wu F , Sheng Y . Social support network, social support, self‐efficacy, health‐promoting behavior and healthy aging among older adults: a pathway analysis. Arch Gerontol Geriat. 2019;85:85103934. 10.1016/j.archger.2019.103934 31466024

[21] Widmer ED , Girardin M , Ludwig C . Conflict structures in family networks of older adults and their relationship with health‐related quality of life. J Fam Issues. 2018;39(6):1573–97. 10.1177/0192513x17714507 29593370 PMC5846743

[22] Xu AQ , Xia Y . The changes in mainland Chinese families during the social transition: a critical analysis. J Comp Fam Stud. 2014;45(1):31–4. 10.3138/jcfs.45.1.31

[23] Hetherington EM . Coping with marital transitions: a family systems perspective. Monogr Soc Res Child Dev. 1992;57(2–3):1–14. 10.1111/j.1540-5834.1992.tb00300.x

[24] Chen G , Gully SM , Eden D . Validation of a new general self-efficacy scale. Organ Res Methods. 2001;4(1):62–83.

[25] Xiao F , Yi CX . A study of the reliability and validity of the General Self‐Efficacy Scale (NGSES). Journal of Mudanjiang Normal University (Philosophy and Social Science Edition). 2012;4:127–129.

[26] Kangwanrattanakul K . A comparison of measurement properties between UK SF‐6D and English EQ‐5D‐5L and Thai EQ‐5D‐5L value sets in general Thai population. Expert Rev Pharmacoecono Outcomes Res. 2021;21(4):765–74. 10.1080/14737167.2021.1829479 32981380

[27] Jian X , Dai B . Application of spss23.0 statistical analysis in psychology and Pedagogy. Beijing: Beijing Normal University Press; 2017.

[28] Liu L‐L , Tian L‐M , Guo J‐J . The effect of parent−child relationship on adolescent risk‐taking behavior: a mediated model with moderation. Psychol Dev Educ. 2019;35(2):84–92.

[29] Wang M . Latent variable modeling and Mplus application. Chongqing: Chongqing University Press; 2014.

[30] Wen Z , Ye B . Intermediary effect analysis: method and model development. Adv Psychol Sci. 2014;5:731–45. 10.3724/SP.J.1042.2014.00731

[31] Nguyen HC , Nguyen MH , Do BN , Tran CQ , Nguyen TTP , Pham KM , et al. People with suspec1ted COVID‐19 symptoms were more likely depressed and had lower health‐related quality of life: the potential benefit of health literacy. J Clin Med. 2020;9(4):965. 10.3390/jcm9040965 32244415 PMC7231234

[32] Slivjak ET , Fishbein JN , Nealis M , Schmiege SJ , Arch JJ . Cancer survivors' perceived vulnerability to COVID‐19 and impacts on cognitive, affective, and behavioral responses to the pandemic. J Psychosoc Oncol. 2021;39(3):366–84. 10.1080/07347332.2021.1887430 33886442 PMC8788202

[33] Tong KK , He M , Wu AMS , Dang L , Chen JH . Cognitive factors influencing COVID‐19 vaccination intentions: an application of the protection motivation theory using a probability community sample. Vaccines. 2021;9(10):1170. 10.3390/vaccines9101170 34696278 PMC8537765

[34] Hashimoto A , Sonohata M , Mawatari M . The use of oral analgesics and pain self‐efficacy are independent predictors of the quality of life of individuals with rheumatoid arthritis. Pain Res Manag. 2020;2020:20207409396. 10.1155/2020/7409396 PMC7396007 32774569

[35] Booysen F , Botha F , Wouters E . Conceptual causal models of socioeconomic status, family structure, family functioning and their role in public health. BMC Public Health. 2021;21(1):191. 10.1186/s12889-021-10214-z 33478444 PMC7821511

[36] Kim YP , Kim S , Joh JY . Family adaptability and cohesion in families consisting of Asian immigrant women living in South Korea: a 3‐year longitudinal study. Asia‐Pacific Psychiatry. 2015;7(2):206–14. 10.1111/appy.12028 23857754

[37] Hong XM , Liu QQ . Parenting stress, social support and parenting self‐efficacy in Chinese families: does the number of children matter? Early Child Dev Care. 2021;191(14):2269–80. 10.1080/03004430.2019.1702036

[38] Segrin C , Flora J . Family communication. New York: Routledge; 2018.

[39] Schippers A , van Boheemen M . Family quality of life empowered by family‐oriented support. J Policy Pract Intellect Disabil. 2009;6(1):19–24. 10.1111/j.1741-1130.2008.00195.x

[40] Weiser DA , Riggio HR . Family background and academic achievement: does self‐efficacy mediate outcomes? Soc Psychol Educ. 2010;13(3):367–83. 10.1007/s11218-010-9115-1

[41] Liang CC , Yuan YH . Exploring children's creative self‐efficacy affected by after‐school program and parent−child relationships. Front Psychol. 2020;11:112237. 10.3389/fpsyg.2020.02237 PMC7522334 33041895

